# On a Means of Suppressing Quackery

**Published:** 1802-11-01

**Authors:** 

**Affiliations:** London


### *To the Editors of the Medical and Physical Journal.*

Gentlemen,

Many judicious and pertinent observations have appeared in several Numbers of your respectable Journal, on the baneful and ignoble practice of Quackery; but I have not observed any plan proposed by those correspondents for its entire suppression, or cause assigned for the encouragement it has met with in this country *particularly*.

Having repeatedly witnessed the injurious effects of several nostrums daily recommended in the papers, as curative of every disease incident to the human frame; the subject has since appeared to me of so great importance, as to induce me to devote much time to the investigation, not only of their compositions, but the abilities of the proprietors, and the testimonies published of their efficacy, the result of which I purpose laying before the public, in a Treatise, entitled, "An Analysis of Quack Medicines, comprising a faithful account of every advertised medicine sold in the united kingdom, the names of the proprietors, their education and origin, the testimonies of cures and authenticated cases, of their baneful effects, &c." In which I shall satisfactorily prove, that this practice is the greatest imposition ever practised in any enlightened country. Many of these *wonderful discoveries* I find to be pharmaceutical preparations sold under a fictitious name, as the balsam or essence of some celebrated herb or medicine. Surely, if impositions are cognizable by our our laws, this fpecies, which affects the health and life of his Majefty's fubjects, is particularly worthy the notice of the legiflature. To my aftonifhment I difcover many of their *innocent* preparations to contain mercury, although they folemnly declare not a particle enters the compofition. Spirit of turpentine diluted with an expreffed oil, is fold under a *pompous title*, as a certain remedy for chronic and *inflammatory* rheumatifm, gout, pleurify, inflammation of the lungs, indigeftion, flatulency, &c. which, I think your readers will agree with me, do not require a fimilar treatment; but, on the contrary, fuch a medicine in inflammation of the lungs, muft be productive of very ferious mifchief. The clafs of nervine medicines, which are fpirit impregnated with the effential oil of vegetables, are merely employed as vehicles for the exhibition of more potent compofitions which are kept in every apothecary's fhop. Hence, in weaknefs of the ftomach, they are directed to be taken with a dofe of Huxham's tincture of bark, in hyfteric fits in conjunction with the fœtid pills, in fuppreffion of the menfes with the pilul. aloet. c. ferro, &c.

In order to render this work as copious and effectual as poffible, in expofing in its true light the practice of thofe felf-created doctors, &c. I fhall be greatly obliged to your readers to favour me with fuch information connected with the fubject, as they may judge likely to promote my views, particularly of their inefficacy or pernicious effects, which muft have occurred in the practice of every medical gentlemen; and as I have already been at a very confiderable expence in purchafing the medicines for examination, their different publications, and in obtaining other information from thofe who have attefted the cures that occafionlly appear in the daily papers, (which are chiefly by their printers and people interefted in the fale of their trafh), and more particularly as I undertake it more for the good of the commonwealth than any emolument that can arife from its publication; I muft requeft, that what communications they may be pleafed to favour me with, may be fent free of expence, addrefsed to C. M. at Mr. Slower's, printer, No. 1, Grange Court, Carey Street, with real fignatures and place of refidence.

Although I fubfcribe myfelf for the prefent under a fictitious name, my real name, with thofe of the chemifts that have affifted me in the examination and decompofition of the medicines, are in the title page of the work. I am, &c.

*London, Sept.* 12, 1802. CHEMICO-MEDICUS.

CRITICAL

